# Correction to: Comparative genomics of *Alternaria* species provides insights into the pathogenic lifestyle of *Alternaria brassicae* – a pathogen of the *Brassicaceae* family

**DOI:** 10.1186/s12864-020-6504-5

**Published:** 2020-01-28

**Authors:** Sivasubramanian Rajarammohan, Kumar Paritosh, Deepak Pental, Jagreet Kaur

**Affiliations:** 1 Department of Genetics, University of Delhi, South Campus, New Delhi, 110021 India; 2 Present Address: National Agri-Food Biotechnology Institute, Mohali, India; 3 Centre for Genetic Manipulation of Crop Plants, University of Delhi South Campus, New Delhi, India

**Correction to: BMC Genomics (2019) 20:1036**

**https://doi.org/10.1186/s12864-019-6414-6**

Following the publication of this article [1], the authors reported that the captions of Figs. 2 and 3 were published in the incorrect order, whereby they mismatch with their corresponding images. The figures are reproduced in the correct sequence with the correct captions in this Correction article. The original article has been corrected.

**Fig. 2:**
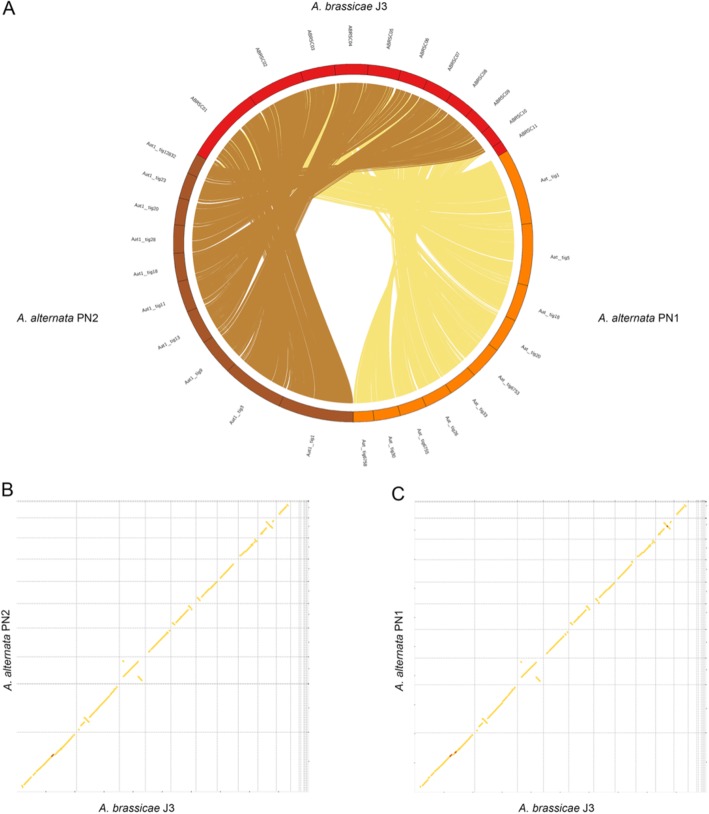
Whole-genome alignments of *A. alternata* PN1 and PN2 with *A. brassicae*. **a** Circos plot showing macrosynteny of *A. alternata* PN1 and PN2 with *A. brassicae* across all contigs except the dispensable contigs (ABRSC11, scaffold13,17,18,19), **b** and **c** Syntenic dotplots of *A. brassicae* with *A. alternata* PN1 and PN2

**Fig. 3:**
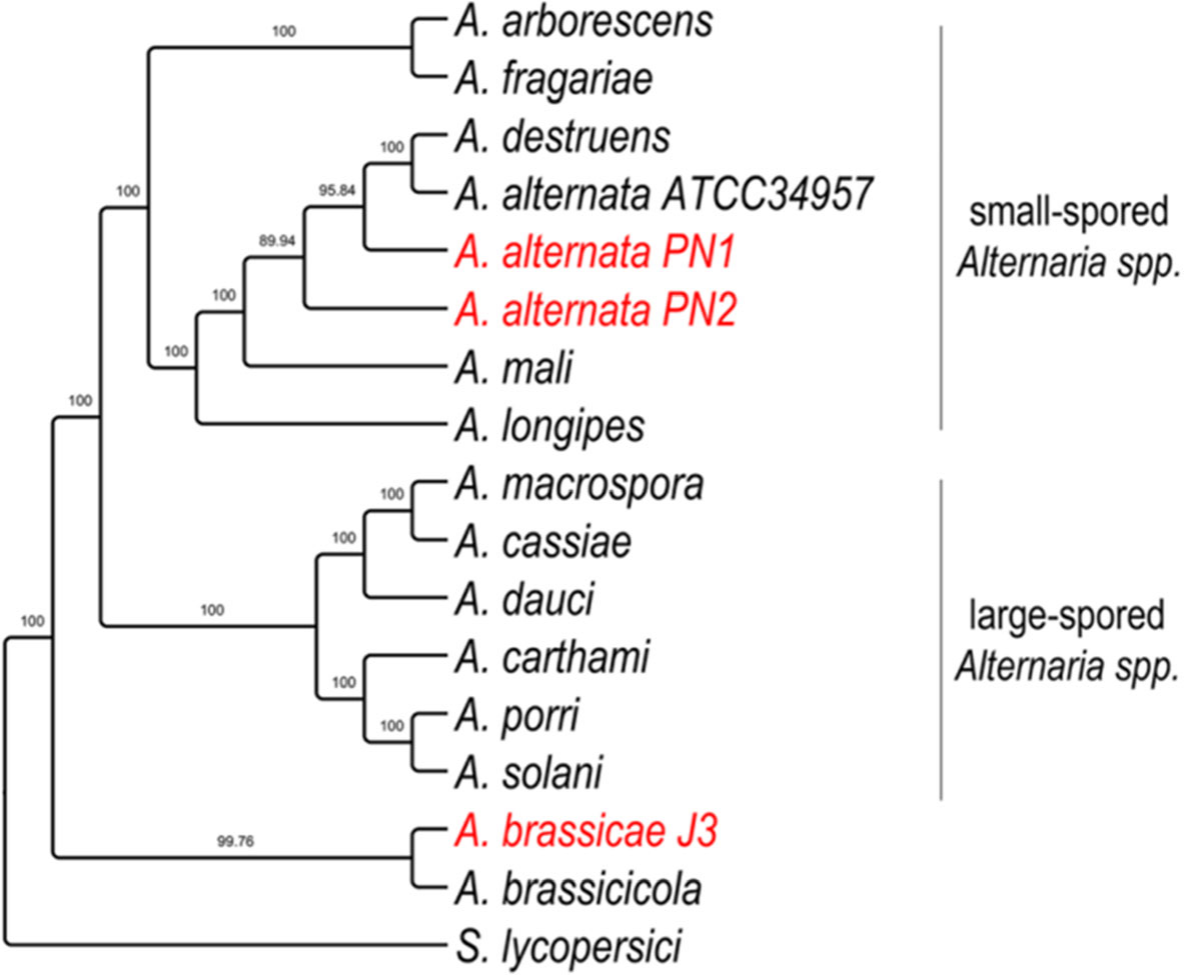
Phylogenetic tree of Alternaria species with S. lycopersici as an outgroup. The tree was constructed using 29 single copy orthologs, which had the highest phylogenetic signal as calculated in Mirlo. Branch support values from 1000 bootstrap replicates are shown
